# 

**Published:** 1838-10-01

**Authors:** 


### BIBLIOGRAPHICAL RECORD.

1. The Transactions of the Provincial, Medical, and Surgical Association, instituted in 1832. Vol. VI. Part 2. 1838.

---

2. Topography of Assam. By John M'Cosh, Assist. Surgeon, Calcutta, 1837.

---

3. A Treatise on Physical Education, specially adapted to Young Ladies. By A. M. Bureaud Riofrey, M.D. Second Edition, greatly enlarged. Longman & Co. June, 1838.

---

4. First Principles of Medicine. By Arch. Billing, M.D. &c. &c. Third Edition, enlarged and improved. Highley, 1838.

---

5. Practical Observations on Hysteria, &c. By John Prichard, M.R.C.S. 8vo, sewed. Churchill, June 1838.

---

6. The Physiognomy of Mental Diseases. By Alex. Morison, M.D. Nos. 1, 2, & 3, price 3s. 6d. each.

☞ *This is a very ingenious and meritorious work.*

---

7. A practical Compendium of the Materia Medica, with numerous Formulæ adapted to the Treatment of Diseases of Infancy and Children. By Alex. Ure, M.D. M.R.C.S. Duodecimo, pp. 236. Schloss, 1838.

---

8. A Dictionary of Practical Medicine, &c. &c. By James Copland, M.D. F.R.S. &c. Part 5. 1838. Price 4s. 6d. June, 1838.

---

9. The Cyclopædia of Practical Surgery, &c. &c. Edited by W. B. Costello, M.D. Part III. July, 1838.

---

10. A Letter respecting Santa Cruz, as a Winter Residence for Invalids. By Joseph Tuckerman. Boston, 1837.

---

11. The Life of Edward Jenner, M.D., with Illustrations of his Doctrines, and Selections from his Correspondence. By John Baron, M. D. In two Volumes, 8vo. Henry Colburn, 1838.

12. Statistical Report on the Sickness, Mortality, and Invaliding among the Troops in the West Indies. Prepared from the Records of the Medical Department and War-Office Returns.

---

13. The third Fasciculus of Anatomical Drawings, selected from the Collections of Morbid Anatomy in the Army Medical Museum at Chatham. Drawn on Stone by Waterhouse Hawkins. Folio, 1838.

---

14. A Catalogue of the Collections of Mammalia and Birds in the Museum of the Army Medical Department at Fort Pitt, Chatham. 1838.

---

15. Appendix to the Catalogue of the Library of the Army Medical Department, in continuation. 1838.

---

16. A Fact in the Natural History of Children, hitherto unobserved, which explains much concerning Infantile Diseases and Mortality. By John Gardner, Surg. Octavo, pp. 23. 1838.

---

17. Remarks on the Preservation of the Teeth, from Infancy to Old Age. By D. W. Jobson, Surgeon-Dentist, &c. Octavo, pp. 94. Hill, 55, Great Windmill Street. Price One Shilling.

---

18. Elements of Chemistry, including the Application of the Science in the Arts. By Thomas Graham, F.R.S. L. et Ed. Professor of Chemistry in the London University College, &c. &c. Part the Second, containing Nomenclature, Notation, and Principles of Chemical Philosophy. Price Two Shillings. Bailliere, July 1838.

---

19. Observations on the Management of Mad-houses; illustrated by Occurrences in the West Riding and Middlesex Asylums. By Caleb Crowther, M.D., formerly Senior Physician to the West Riding Pauper Lunatic Asylum. Octavo, pp. 145. Simpkin and Marshall, 1838.

---

20. Medico-Chirurgical Transactions, published by the Royal Medical and Chirurgical Society. Vol. 21. July 1838.

21. A Treatise on Inflammation. By James Macartney, M.D. &c. &c. In 4to, pp. 214, with Plates. Longman and Co. 1838.

---

22. Medical Portrait Gallery. Part VI. Price 3s. By T. J. Pettigrew, Esq.,—Sir A. Cooper, Dr. J. Copland, Dr. J. Cooke, Mead.

---

23. Counter-irritation; Principles and Practice. Illustrated by One Hundred Cases of the most painful and important Diseases effectually cured by external Applications. By A. B. Granville, M.D. &c. &c. 8vo, pp. 353. Churchill, 1838.

---

24. The Cyclopædia of Anatomy and Physiology. Edited by Dr. Todd, &c. Part XV. Containing—Gland, by Mr. Grainger—Glosso-pharyngeal Nerve, by Dr. Reid—Gluteal Region, by A. T. S. Dodd, Esq.—Hæmatosine, by Dr. G. O. Rees—Hand, by Dr. Todd, Mr. Adams, and Mr. M'Dougall—Hearing, by T. W. Jones, Esq. Illustrated wish numerous Engravings. August, 1838.

---

25. Eighteenth Report of the Directors of the Dundee Lunatic Asylum, for the Year ending 31st May, 1838.

---

26. Elements of Physiology; being an Account of the Laws and Principles of the Animal Economy, especially in reference to the Constitution of Man. By Thomas Johnstone Aitkin, M.D. &c. &c. Small octavo, pp. 514. Scott, Webster, and Geary, London, Aug. 1838.

---

27. The Science of the Cerebro-spinal Phenomena attempted. By John S. Waugh, M.D. Annan. Small octavo. S. Highley, 1838.

---

28. The Structure, Diseases, and Treatment of the Teeth, considered with a View to the Abolition, in all common cases, of the pernicious Practice of Tooth-drawing. By W. Wardroper, M.R.C.S. Octavo, pp. 59. Renshaw, London, Aug. 1838.

---

No. 4, price 3s. 6d. the Physiology of Mental Diseases. By Sir Alex. Morison, M.D.

☞ *This number contains five striking Portraitures of Monomania, chiefly females.*

---

30. A Treatise on the Nature and Treatment of Hooping-cough and its Complications, illustrated by Cases; with an Appendix, containing Hints on the Management of Children, &c. &c. By George Hamilton Roe, M.D. Physician to the Westminster Hospital. Octavo, pp. 258. Churchill, 1838.

---

31. Recherches Medico-physiologiques sur l'Electricité Animal, &c. &c. &c. Par J. F. Condret, M D. Octavo, Paris, 1837.

---

32. Tables, shewing Thermometrical Results in the Months of January & February, 1838, at Torquay, Exeter, Cheltenham, Bristol, London, Edmonton, Sheffield, &c. By R. F. De Barry, M.D.

---

33. Experimental Essay on the Physiology of the Blood, for which the Gold Medal was awarded by the Medical Faculty of the University of Edinburgh. By Chas. Maitland, M.D. Brighton. Octavo, pp. 90. Carfrae & Son, Ed. 1838.

☞ *In our next.*

---

34. A History of British Birds. By William Yarrell, F.L.S. &c. Illustrated by a Wood-cut of each Species, and numerous Vignettes. Part VIII. price 2s. 6d. Van Voorst, Paternoster Row, 1838.

---

35. A General Outline of the Animal Kingdom. By Thomas Rymer Jones, F. Z. S., Professor of Comparative Anatomy in King's College, London. Illustrated by numerous Engravings on Wood. Part I. price 2s. 6d. Van Voorst, 1838.

---

36. A Treatise on Neuralgia. By Rich. Rowland, M.D. Physician to the City Dispensary. Sept. 1838. Highley.

☞ *Too late for this number.*

---

37. Analytical Tables; adapted to the Purpose of General and Forensic Chemistry; with Directions for preparing the various Tests, and a Description of the Means of ascertaining their Purity, &c. Translated from the French of Professor Devergie. By Martin H. Lynch, M.D. Lecturer in the Newcastle-on-Tyne School of Medicine, &c. Nos. 1 and 2.

⁂ *These Tables or Charts are very ingeniously planned, and must prove very useful both for reference and study.*

---

38. On Urinary Diseases; the Consequences and Treatment. By Robert Willis, M.D. Licentiate of the Royal College of Physicians, &c. Octavo, pp. 408. With Lithographic Plates. October, 1838.

### TO CORRESPONDENTS.

We had prepared a long analysis of the Army Medical Reports from the West Indies, but a pressure of other matter has compelled us to postpone its insertion till next number. We did not deem it necessary to postpone Dr. Fergusson's memorandum on the same subject.

*To the Medical Officers of Hospitals and Infirmaries.*

As we now dedicate two or three sheets of Extra-Limites quarterly to the publication of select Clinical Reports and important cases, a favorable opportunity is afforded to hospital physicians and surgeons, to make known their experience, both in this country and America. The Reports are requested to be forwarded as early in the quarter as possible.

Dr. Sharkey's paper on the epidemic cholera of last year in the South-west of Ireland, is received, and under consideration.

We have to apologize for the omission of several articles this number. They will appear in our next.

### BIBLIOGRAPHICAL RECORD, *Continued.*

1. The Naturalist's Library. Conducted by Sir WILLIAM JARDINE, Bt. Vol. VII. British Quadrupeds. By William Macgillivray, A.M. F.R.S. &c. Edinburgh, Lizars; London, Highley. Price 6s. 1838.

2. An Essay on Food. By W. GRISENTHWAITE, Author of a Refutation of Paine's "Age of Reason," New Theory of Agriculture, &c. Small 8vo, pp. 119. W. Crofts, London, Sept. 1838.

3. The Phrenological Journal. No. IV. New Series, for October, 1838.

4. A System of Practicai Surgery, with numerous explanatory Plates, the Drawings after Nature. By JOHN LIZARS, Professor of Surgery, &c. &c. Octavo, pp. 220, Eighteen Plates. Highley, October, 1838. Price 10s. 6d.

5. The India Review, and Journal of Foreign Science and the Arts. Edited by FRED. CORBYN, Esq. Nos. 23 and 24, for March and April, 1838.—*In exchange.*

6. The India Journal of Medical and Physical Science. Edited by FRED. CORBYN, Esq. New Series. Nos. 3 & 4. March and April, 1838.—*In exchange.*

7. Dental Practice, or, Observations on he Qualifications of the Surgeon-Dentist —Dental Quackery—Nature and Extent of the Duties of the Dentist in the first and second Dentition. The defective Teeth of the present day compared with those of the last century, owing to Empiricism—Regulation and Management of the Teeth—The present absurd and destructive practice of filing Teeth—Treatment of Toothache—Extraction of Teeth—New extracting Instruments invented by the Author, illustrated by plates—Third Dentition—Importance of Artificial Teeth—Philosophical Principles on which they are formed —And on the Duty of the Surgeon-Dentists and Mechanical Dentists, each by themselves, to unite in associations, for the purpose of abating dental Quackery. By JOHN GRAY, Surgeon-Dentist, Member of the Royal College of Surgeons in London. 12mo, pp. 54. London, 1837.

8. Anatomical Tables, containing Descriptions of the Muscles, Ligaments, Fasciæ, Blood-vessels, and Nerves. Intended for the Use of Students. By THOMAS NUNNELEY, Lecturer on Anatomy and Physiology in the Leeds School of Medicine, &c. &c. 18mo, pp. 240. London, 1838.

9. Chemistry of Organic Bodies. Vegetables. By THOMAS THOMSON, M.D. Regius Professor of Chemistry in the University of Glasgow, &c. &c. Octavo, pp. 1068. Bailliere, London, 1838.

### INDEX.

A.

Abdominal tumors, Dr. Bright on.... 211
Abdominal tumors, observations on.. 211
Abortion, causes of ............... 242
Abscess near the anus ............ 1
Abscesses independent of diseases of the rectum .................... 1
Abscesses arising from a morbid condition of the rectum ............ 4
Acupuncturation, cure of hernia by.. 256
Acute pericarditis, complicated with pleurisy ..................... 344
Acute purulent inflam. of conjunctiva, 363
Acute pericarditis, case of.......... 341
Acute rheumatism, Dr. Seymour on.. 657
Acute fibrous rheumatism.......... 657
Addenbrooke Hospital ............ 320
Affections of the uterus, use of arsenic in 370
Ague, complication of, with ascites .. 601
Air, introduction of, into the veins .. 252
Air, Velpeau on the introduction of, into the veins .................. 574
Albuminous fluids, Dr. Babington on, 609
Albuminous urine of renal dropsy ... 611
Alternative, by John Pinney, Esq. .. 201
Amaurosis, treatment of ........... 627
Amenorrhœa, treatment of ........ 622
Amputation, phlebitis after ........ 259
Anatomical plates, by Jones Quain .. 209
Anatomical Diagrams, by T. Wormauld 210
Anatomical tables ................ 547
Anatomy of the teeth, M. Blandin on, 38
Anatomy of regions, by Velpeau .... 209
Anatomy of regions, by Dr. Lebaudy, 209
Anemia ......................... 98
Aneurysm ...................... 448
Aneurysm, operation for .......... 449
Aneurysm, brachial .............. 664
Angeioleucitis .................... 452
Angina .......................... 579
Animal heat ..................... 439
Animal magnetism, Baron Duptotet on 59
Animal magnetism again .......... 285
Animal magnetism................ 634
Animalculæ in the human blood .... 225
Anus, contraction of the .......... 17
Apoplexy & epilepsy, relation between 638
Application of arithmetic to therapeut. 598
Argenti nitras.................... 539
Arithmetic, application of, to therapeut. 598
Arm-drop, case of ................ 297
Arsenic, use of, in affections of uterus 370
Artery, ulnar, case of wounded .... 301
Articular rheumatism ............. 581
Artificial digestion, Dr. Parkinge on.. 114
Artificial digest. of coagulable albumen 120
Artificial digestion, influence of galvanism in ..................... 114
Ascites, complication of ague with .. 601
Ashwell on hæmorrhage from the unimpregnated uterus ............. 615
Aura menstrualis, observations on .. 607
Auscultatory sounds in large arteries, 569

B.

Babington on albuminous fluids..... 609
Bandages, M. Gerdy on............ 248
Baron's life of Jenner.............. 488
Beaumont on the gastric juice ...... 205
Bed-sores, useful application to...... 272
Bell's history of British reptiles ..... 207
Belladonna in puerperal mania ...... 556
Billing's first principles of medicine.. 541
Birds, British, Yarrell's history of .. 207
Birds, ventriloquism in ............ 565
Birmingham Infirmary, report of out-patients of ..................... 619
Birmingham Dispensary, report of the 622
Birmingham Eye Infirmary, report of, 624
Bismuth, subnitrate of, in gastralgia.. 580
Bite of a cobra capella ............ 282
Bladder, Mr. Coulson on diseases of, 166
Bladder, irritability of the.......... 166
Bladder, paralysis of the .......... 172
Bladder, inflammation of mucous membrane of the .............. 175
Bladder, subacute inflammation of mucous membrane of the .......... 178
Bladder, acute inflam. of muscular coat 181
Bladder, chronic inflammation of muscular coat of the................ 183
Bladder, inflam. of peritoneal coat of, 184
Bladder, fungous hæmatodes of the.. 185
Bladder, cancer of the ............ 185
Bladder & rectum, purulent disch. from 306
Blandin on the anatomy of the teeth, 38
Blandin on phlebitis .............. 592
Blepharitis, Velpeau on treatment of, 591
Blepharitis, mucous .............. 591
Blepharitis, glandular.............. 591
Blepharitis of new-born infants...... 592
Blisters, use of, in erysipelas of scalp, 246
Blood, animalculæ in the ......... 225
Blood, Mitscherlich on the changes of, 561
Boivin on the causes of abortion .... 242
Bostock on the constitution of urine, 383
Boucheron on the hair ............ 249
Bouillaud's clinique .............. 578
Brachial aneurysm................ 664
Brain, signs of undue development of, 538
Brigham on the influence of religion on health...................... 529
Bright on abdominal tumors........ 211

British birds, Yarrell's history of .... 207
British reptiles, Bell's history of .... 207
Bronchitis .................... 579
Bronchitis .................... 333
Bronchocele removed by croton oil .. 281
Bronchotomy, Dr. Johnson's case of, 278
Bushe on diseases of the rectum .... 1
Buxton waters, Dr. Robertson on the, 531
C.
Calcutta, medical topography of .... 190
Calcutta naval hospital ........... 319
Calcutta, transactions of the medical and physical society of .......... 405
Cancer of the bladder.............. 185
Cancer of the face ................ 356
Carcinomatous degeneration of rectum 31
Carmichael on sciatica .......... .. 198
Caspar on the duration of human life, 250
Cataract, case of.................. 304
Causes of sudden death ............ 270
Causes of insanity ................ 413
Cervical vertebra, fracture of ....... 302
Cervix uteri, simple ulceration of the, 433
Cervix uteri, excision of the........ 436
Cervix uteri, inflammation of the.... 426
Changes in the blood, Mitscherlich on, 561
Chemical nature of mucous and purulent secretions.................. 611
Chemistry of organic bodies ........ 548
Cholera, malignant, in the Dreadnought 386
Cholera, infection of .............. 390
Chomel on tartar-emetic in pneumonia 594
Chorea.......................... 665
Chronic vomiting ................. 582
Chronic diseases, nitrate of silver in.. 554
Churchill on diseases of females .... 417
Churchill's medical report..... .... 631
Circulating system, homicide by injuries of the.................... 160
Clavicle, removal of the............ 373
Climate and diseases of Swan River.. 281
Climate, influence of, on man ...... 437
Climate of Torquay ................ 554
Clinical medicine, M. Rostan on .... 597
Clinical report of Philadelphia hospital 629
Clinique of M. Bouillaud ........... 578
Cobra capella, bite of a ............ 282
Cold climates, diseases of .......... 443
Colquhoun's case of exten. liver abscess 409
Compendium of the Materia Medica, by Dr. Ure .................... 539
Complication of fistula with stricture, 31
Compression of blood-ves. in neuralgia 241
Conjunctiva, acute purul. inflam. of.. 363
Constitutional hæmorrhage, remarks on 227
Constitution of urine, Dr. Bostock on, 383
Contagion of the variola............ 240
Contraction of the anus............. 17
Cooper, Sir A. on varicocele of the spermatic cord ................. 612
Cornea, removal of opacity of the.... 409
Cornea, treatment of staphyloma of .. 625
Corroding ulcer of the uterus........ 435
Cosmetics, pernicious effects of...... 584
Coulson on diseases of the bladder .. 166
County Clare Infirmary, cases from.. 660
Cove, medical topography of ........ 276
Counter-irritation, Dr. Granville on, 471
Criminals, reform of .............. 191
Croton oil, removal of bronchocele by 281
Crowther on managem^t. of madhouses 542
Curvature, lateral, of the spine...... 303
Cutaneous absorption, Dr. Madden on 187
Cyclopædia of practical surgery .... 448
D.
Depressed fracture of skull......... 660
Diabetes mellitus, Dr. Johnson's case, 552
Dickson on dropsy ................ 289
Dickson, Dr., letter ............... 674
Dicksonian finish ................. 283
Diffuse popliteal aneurysm, case of .. 297
Digestion, Beaumont on............ 205
Diseases of the rectum ............ 1
Diseases of the rectum, Mr. Syme on, 1
Diseases of the rectum, Dr. Bushe on, 1
Diseases of the ear, Kramer on ...... 129
Diseases of the internal ear ........ 140
Diseases of the bladder, Mr. Coulson on 166
Diseases of the kidneys, M. Rayer on, 234
Diseases of the pulmonary organs.... 322
Diseases of the heart .............. 339
Diseases of cold climates............ 443
Diseases of females, Dr. Churchill on, 417
Diseases arising from insects........ 562
Diseases of old age ................ 597
Dislocation of the humerus ........ 409
Dislocation of the lens ............ 626
Dreadnought, malig. cholera on board, 386
Dropsy, Sir David Dickson on ...... 289
Dropsy of the mucosæ, treatment of.. 589
Dropsy of the joints ............... 590
Dumfries and Galloway infirm. report 293
Dupotet on animal magnetism ...... 59
E.
Ear, Kramer on diseases of the ...... 129
Ear, case of wound of the .......... 246
Ectopia cordis, and umbilical hernia, Dr. O'Brien's case of ............ 399
Effect upon pulse by change of posture 615
Ellis on insanity .................. 122
Emmenagogues, observations on .... 607
Endo-carditis, case of.............. 343
England on gangrene of the lungs.... 397
Enlarged liver, Mr. Malcolmson on .. 360
Enlargements of the spleen, quinine in 233
Entozoa in the human body ........ 565
Epilepsy, treatment of, with nitrate of silver ........................ 603
Epilepsy & apoplexy, relation between, 638
Erections of the penis ............. 567
Ergot of rye in paralytic affections .. 242
Erysipelas of the scalp, use of blisters in 246

Erysipelas ........................ 457
Esquirol on mental disorders........ 410
Excision of the lower maxilla........ 376
Extension of the reflex motions...... 145
Extension in muscular contractions .. 260
Extirpation of the eye.............. 403
Eye, extirpation of the ........... 403
F.
Face, malignant diseases of the skin of 355
Face, cancer of the................ 356
Face, fungous cancer of the ........ 358
Fallopian tubes, inflammation of the, 431
Females, Dr. Churchill on diseases of, 417
Femoral hernia, case of............ 296
Femur, fracture of the ............ 302
Firelock, wounds from bursting of a.. 304
First princip. of medicine, Dr. Billing's, 541
Fistula in ano.................... 1
Fistula, symptoms of.............. 9
Fistula, treatment of .............. 11
Fistula, complication of, with phthisis 14
Fistula, complication of, with stricture 31
Fluids, albuminous, Dr. Babington on, 609
Fluid in vesiculæ seminales of man .. 558
Foissac on influence of climate on man 437
Foreign bodies in the bladder........ 185
Forensic medicine ................. 152
Fracture of the patella, transverse .. 302
Fracture of the femur.............. 302
Fracture of cervical vertebra ....... 302
Fractured phalanges, case of ........ 301
Fractures, Mr. Lonsdale on ........ 204
Fractures, Velpeau on the treatment of 247
Frogs, state of, after decapitation.... 142
Fungus hæmatodes of the bladder.... 185
G.
Galvanism, influence in artificial digest. 114
Gangrene of the lungs ............ 231
Gangrene of the lungs, Dr. England on 397
Gangrene of the leg................ 663
Gardner on kephalosis ............ 537
Gastralgia, subnitrate of bismuth in.. 580
Gastric juice, Mr. Beaumont on the .. 205
Generation, signs from the parts of .. 485
Geneva hospital, report of the ...... 628
Gerdy on bandages................ 248
German journals, observations from.. 581
Glanders in the human subject...... 239
Gleanings in the Indian seas ........ 666
Glottis, spasm of the .............. 228
Granville on counter-irritation...... 471
Gray's, Dr. utero-abdominal supporter 550
Great sympathetic, Dr. Segond on neuralgia of the.................... 462
Guy's Hospital .................... 609
H.
Hæmorrhage, constitutional remarks on 226
Hæmorrhage from unimpreg. uterus, 615
Hair, M. Boucheron on the ........ 249
Halford, Sir Henry, life of.......... 193
Hawkins on malignant diseases of skin of the face .................... 355
Heart, diseases of the............. 339
Heart, rheumatic affections of .. 239, 345
Heart, diseases of the, not rheumatic, 346
Heart, hypertrophy of the.......... 351
Heart, hypertrophy of, Mr. Salter on, 395
Heart, Stephen's case of rupture of.. 671
Hernia, Mr. Hilles on ............ 203
Hernia, cure of, by acupuncturation.. 256
Hernia, liabilities to .............. 560
Hilles on hernia .................. 203
History of the anatomy of the teeth.. 39
History of semeiology.............. 481
Homicide, Mr. Watson on .......... 152
Homicide, general remarks on ...... 155
Homicide by injuries of nervous system 158
Homicide by injuries of circulat. system 160
Homicide by injuries of respir. system 162
Homicide by injuries of nutrat. system 164
Hooping-cough treated with carb. iron 265
Hooping-cough, Dr. Roe on........ 545
Hooping-cough .................. 581
Human subject, glanders in the .... 239
Human life, Dr. Caspar on duration of 250
Humerus, dislocation of the ........ 490
Hunter's works .................. 38
Hydatid of the liver .............. 368
Hydrocele, cases of................ 319
Hydrocephalus, tapping the head in.. 273
Hypertrophy of the heart ....... .. 351
Hypertrophy of the heart, Mr. Salter on 395
Hysteria, Mr. Prichard on.......... 534
Hysteria, singular case of .......... 586
I.
Idiopathic abscesses ............... 2
Indian seas, gleanings in the ........ 666
Infant, life and viability of an ...... 576
Inflammation of the conjunctiva, Mr. Tyrrell on ...................... 363
Inflammat$^{n}$. of deep-seated lymphatics 454
Inflammation of unimpregnated uterus 430
Inflammation of fallopian tubes ..... 431
Inflammation of ovaries............ 432
Inflammatory affections of the stomach 104
Influence of stomach on other organs, 106
Influence of climate on man ........ 437
Influence of religion on health ...... 529
Injuries of the spinal cord .......... 158
Insane, paralysis in the... ......... 595
Insanity, Sir W. C. Ellis on ........ 122
Insanity, nature of................ 122
Insanity, treatment of ............ 126
Insanity, moral treatment of........ 127
Insanity, symptoms of ............ 411
Insanity, causes of................ 413
Insects, Raspail on diseases arising from 562
Insects, temperature of ............ 565
Internal ear, diseases of the ........ 140
Invertebrata, teeth of the .......... 53
Irritability of the bladder .......... 166
Irritability of the bladder, treatment of 169
J.
Jenner, life of..................... 488

Johnson's, Dr case of bronchotomy.. 278
Johnson's, Dr. case of diabetes mellitus 552
Joints, dropsy of the .............. 590

K.

Kephalosis, Mr. Gardner on ........ 537
Kidneys, Rayer on diseases of the.... 234
King on compression of left bronchus, 513
Kramer on diseases of the ear ...... 129

L.

Labia pudendi, phlegmonous inflam. of 420
Land scurvy ..................... 282
Large arteries, auscultatory sounds in 569
Large calculus, account of a........ 614
Laryngitis ........................ 338
Lateral curvature of the spine ...... 303
Lebaudy's anatomy of regions ...... 209
Left bronchus, compression of...... 613
Leg, gangrene of the .............. 663
Lens, dislocation of the ............ 626
Liabilities to hernia .............. 560
Life of Jenner...................... 488
Life and viability of an infant ...... 576
Liver, influence of the stomach on the 106
Liver, chronic enlargement of the .. 190
Liver, enlarged, Mr. Malcolmson on 360
Liver, hydatid of the.............. 368
Liver-abscess, case of.............. 409
London and Paris, variola in ...... 585
Lonsdale on fractures.............. 204
Lower maxilla, excision of the ...... 376
Lungs, gangrene of the ............ 231
Lungs, Dr. England on gangrene of.. 397
Lymphatics, inflammation of the.... 454

M.

Madden on cutaneous absorption.... 187
Mad-houses, Dr. Crowther on the management of ................ 542
Malcolmson on enlarged liver ...... 360
Malcolmson's case of ranula ........ 405
Male and female sexes, relations of the 267
Malignant stricture of the rectum.... 31
Malignant diseases of skin of the face 355
Malignant pustule in man.......... 582
Mammalia, teeth of the............ 56
Man, influence of climate on........ 437
Man, malignant pustule in.......... 582
Management of madhouses, Dr. Crowther on......................... 542
Marriage, philosophy of............ 197
Marshall's gleanings in the Indian seas 666
Martin on med. topography of Calcutta 190
Materia medica, Dr. Ure's compend. 539
Medical jurisprudence, remarks on .. 152
Medical topography of Calcutta...... 190
Medical portrait Gallery............ 192
Medical topography of Cove........ 276
Medical and surgical transactions.... 353
Medical and Surgical Association, Provincial transactions of............ 353
Medical and physical society of Calcutta, transactions of............ 405
Medicine, first principles of ........ 541
Medico-legal definitions of wounds .. 155
Menstruation, researches on........ 605
Menstruation, remarkable case of.... 606
Mental disorders, M. Esquirol on.... 410
Mercury, proto-ioduret of, in syphilis 607
Microscopic examination of the urine 267
Military med. report from West Indies 653
Milligan on climate of Swan River.. 281
Mitscherlich on the changes of blood 561
Monat on purulent discharges ...... 306
Moral treatment of insanity ........ 127
Morbid sensibility of nerves of stomach 102
Morbid diatheses affecting Europe .. 265
Morbid flattening of left bronchus .. 613
Mucous memb. of bladder, inflam. of 175
Mucous membrane of the vulva, inflammation of the.............. 422
Mucous membrane of the vagina, inflammation of the .............. 424
Mucous and purulent secretions, chemical nature of.............. 611
Muscular coat of bladder, acute inflammation of.................. 181
Muscular coat of bladder, chronic inflammation of .................. 183
Muscular contractions, extension in.. 260

N.

Natural history, scientific notices in.. 565
Naval medical officers, regulations for 284
Nerves of stomach, morb. sensibility of 102
Nerves, Hall's division of the ...... 149
Nervous affections of the stomach.... 104
Nervous system, homicide by injuries of 156
Nervous affections peculiar to young women ...................... 377
Neuralgia, compres. of blood-vessels in 241
Neuralgia of the great sympathetic, Dr. Segond on ...................... 462
Neuritis ........................ 457
New South Wales ................ 660
Newry Dispensary, report of the .... 632
Nipples, ulcers on the...... ....... 582
Nitrate of silver, on the use of the .. 233
Nitrate of silver, treat. of epilepsy with 603
Nitrate of silver in chronic diseases .. 554
Nutritive system, homicide by injur. of 164

O.

O'Brien's case of ectopia cordis and umbilical hernia................ 399
Observations from the German Journ. 581
Œsophagus, ulcerated stricture of the 407
Old age, on the diseases of ........ 597
Opacity of the cornea, removal of.... 409
Operation for stone .............. 185
Organic stricture of the rectum .... 19
Organic bodies, chemistry of........ 549
Organization of the teeth.......... 42
Outpatients of Birmingham Infirmary, report of .................... 619
Ovarian tumors .................. 219

Ovarics, inflammation of the ...... 432
P.
Paralysis of the bladder............ 172
Paralysis of the bladder, causes of.... 173
Paralysis in the insane ............ 595
Paralysis of the portio dura .. ..... 662
Paralytic affections, ergot of rye in.. 242
Parker on the stomach ............ 97
Parker on ulceration of the stomach.. 641
Parker's reclamation .............. 653
Pathological semeiology, Dr. Schill on 477
Patella, transverse fracture of the.... 302
Pathology of the supra-renal capsules 237
Penis, erections of the ............ 567
Pericarditis ...................... 395
Peritoneal coat of bladder, inflam. of 184
Pernicious effects of cosmetics...... 584
Pettigrew's medical portrait gallery .. 192
Philadelphia hospital, clinical report of 629
Philosophy of marriage............ 197
Phlebitis after amputation.......... 259
Phlebitis ...................... 456
Phlebitis, case of, after venesection .. 592
Phlegmonous inflam. of labia pudendi 420
Phthisis, complication of fistula with, 14
Phthisis, treatment of.............. 231
Phthisis ........................ 319
Physical education, Mr. Smiles on .. 204
Pinney's alternative .............. 201
Pleuritis ........................ 337
Pneumonia ...................... 335
Pneumonia, on tartar emetic in .... 594
Poisonous effects of rue............ 604
Polypus of the rectum ............ 35
Portio dura, paralysis of the........ 662
Posterior tibial artery, wound of the.. 664
Practical surgery, cyclopædia of .... 448
Priapism in typhus fever .......... 633
Prichard on hysteria .............. 534
Proto-ioduret of iron in syphilis .... 264
Proto-ioduret of mercury in syphilis 607
Provincial medical & surg. association 353
Puerperal mania, belladonna in...... 556
Pulmonary organs, diseases of the .. 322
Pulmonary emphysema ............ 578
Pulse, effect upon, by change of posture 615
Pupil, state of, in typhus .......... 555
Purkinge on artificial digestion.. ... 114
Purulent discharges from bladder and rectum ...................... 306
Q.
Quain's anatomical plates.......... 209
Quinine in enlargements of the spleen 233
R.
Ranula, Mr. Malcolmson's case of .. 405
Raspail on diseases arising from insects 562
Rayer on diseases of the kidneys .... 235
Reclamation of Mr. Parker ...... 653
Rectum, Mr. Syme on diseases of the 1
Rectum, Dr. Bushe on diseases of the 1
Rectum, stricture of the ..... .... 18
Rectum, abscesses arising from the morbid condition of the ........ 4
Rectum, spasmodic stricture of the.. 18
Rectum, organic stricture of the ... 18
Rectum, simple stricture of the .... 21
Rectum, treatment of stricture of the 26
Rectum, malignant stricture of the .. 31
Rectum, carcinomatous degeneration 31
Rectum, polypus of the............ 35
Reflex motions, Dr. Volkman on .... 142
Reflex motions, simplest phenomenon 143
Reflex motions, on dividing the spinal cord longitudinally.............. 144
Reflex motions, determinateness of .. 145
Reflex motions, extension of the.... 145
Reform of criminals .............. 191
Relations of the male and female sexes 269
Relationship between epilepsy and apoplexy ................ ..... 638
Religion, influence of, on health .... 529
Removal of the clavicle .......... 373
Removal of opacity of the cornea.... 409
Renal dropsy, albuminous urine of .. 611
Report of Dumfries & Galloway Infir. 293
Report of out-patients of Birmingham Infirmary .................... 619
Report of the Birmingham Dispensary 622
Report of the Birming. Eye Infirmary 624
Report from St. George's Hospital .. 657
Report of the Geneva Hospital...... 628
Report of the Newry Dispensary .... 632
Researches on menstruation ........ 605
Respiratory system, homicide by injuries of the .................. 102
Re-vaccination, results of, in Silesia.. 588
Rheumatic affections of the heart.... 339
Rheumatism, acute, Dr. Seymour on 657
Robertson on the Buxton waters. .. 531
Roe, Dr. on hooping-cough ........ 545
Rostan on clinical medicine........ 597
Rue, poisonous effects of .......... 604
Rupture of the heart, case of. ...... 671
Ryan's philosophy of marriage...... 197
S.
St. Georges's hospital, medical report 657
Salter on hypertrophy of the heart .. 395
Schill on pathological semeiology .... 477
Sciatica, Dr. Carmichael on ........ 198
Scientific notices in natural history .. 565
Scott's medical topography of Cove.. 276
Segond on neuralgia of the great sympathetic ...................... 462
Semeiology, history of ............ 481
Seymour on acute rheumatism...... 657
Signs of undue development of the brain ...................... 538
Signs from the parts of generation .. 485
Silesia, results of re-vaccination in .. 588
Silver, preparations of, in syphilis .. 608
Skin of the face, malignant diseases of the ...................... 355

Skull, depressed fracture of ........ 660
Smiles on physical education. ...... 204
Spasm of the glottis .............. 228
Spasmodic stricture of the rectum .. 18
Spermatic cord, Sir A. Cooper on varicocele of the ............. .... 612
Spinal cord, injuries of the ........ 158
Spine, lateral curvature of the...... 303
Spleen, quinine, in enlargement of the 233
Staphyloma of the cornea, treatment of 625
State of the pupil in typhus ........ 555
Stephen's case of rupture of the heart 671
Stomach, Mr. Parker on the........ 97
Stomach, nervous affections of the .. 104
Stomach, inflammatory affections of the 104
Stomach, influence of the, on other organs ....................... 106
Stomach, treatment of diseases of the 110
Stomach, Parker on ulceration of the 641
Strangulated hernia, case of ........ 621
Stricture of the rectum............ 18
Stricture of the rectum, organic .... 19
Stricture of the rectum, treatment of 26
Stricture of the rectum, malignant .. 31
Stricture, complication of fistula with 31
Stricture of the œsophagus, ulcerated 407
Strychnine, therapeutic uses of .... 587
Strychnine, action of.............. 587
Subnitrate of bismuth in gastralgia .. 580
Sudden death, causes of............ 270
Sudden death ..................... 572
Supra-renal capsules, patholony of the 237
Swan river, climate of ............ 281
Syphilis, proto-ioduret of iron in.... 264
Syphilis, proto-ioduret of mercury in 607
Syphilis, preparations of silver in.... 608
Syme on diseases of the rectum .... 1
Symptomatic abscesses ............. 3
Symptoms of insanity ............ 411

T.

Tapping the head in hydrocephalus .. 273
Tartar emetic in pneumonia ........ 594
Teeth, anatomy of the ... ......... 38
Teeth, M. Blandin on anatomy of the 38
Teeth, organization of the ........ 42
Teeth, development of the ........ 46
Teeth of the invertebrata ........... 53
Teeth of the vertebrata ............. 54
Temperature of insects ............. 565
Thames improvement company...... 280
Therapeutic uses of strychnine ...... 587
Therapeutics, appln. of arithmetic to 599
Thymic asthma, treatment of ...... 582
Torquay, climate of................ 554
Transactions of the provincial medical and surgical association .......... 353
Transverse fracture of the patella.... 302
Traumatic abscesses ............... 3
Treatment of irritability of the bladder 169
Treatment of phthisis ............. 231
Treatment of dropsy of the mucosæ.. 589
Treatment of blepharitis, M. Velpeau on 591
Treatment of ulceration of the stomach 648
Tyrrell on inflam. of the conjunctiva 363

U.

Ulcer of the uterus, corroding...... 435
Ulcerated stricture of the œsophagus 407
Ulceration, simple, of the cervix uteri 433
Ulceration of the stomach, Mr. Parker on 641
Ulceration of the stomach, symptoms of 645
Ulceration of the stomach, treatment of 648
Unimpregnated uterus, inflammation of 430
Unimpregnated uterus, Ashwell on .. 615
Ure's compendium of the materia medica 539
Urethro-rectal fistula............... 15
Urine, constitution of the.......... 383
Urine, microscopical examination of 267
Useful application to bed-sores...... 272
Uterine leucorrhœa .............. 427
Utero-abdominal supporter, Dr. Gray's 550
Uterus, use of arsenic in affections of 371
Uterus, corroding ulcer of the ...... 435
Uterus, inflammation of the mucous membrane of the .............. 427

V.

Vagina, inflammation of the mucous membrane of the ............. 424
Varicocele of the spermatic cord, Sir A. Cooper on .................. 612
Variola, contagion of the . ......... 240
Variola in London and Paris........ 585
Veins, introduction of air into the .. 252
Veins, Velpeau on the introduction of air into the .................. 574
Velpeau's anatomy of regions ...... 209
Velpeau on the treatment of fractures 247
Velpeau on the introduction of air into the veins ..................... 574
Velpeau on the treatment of blepharitis 591
Ventriloquism in birds ........... 565
Vertebrata, teeth of the............ 5[illegible]
Vesico-vaginal fistulæ, new instrument for ........................ 383
Vesiculæ seminales, fluid in the .... 558
Volkman on reflex motions ........ 142
Vulva, inflammation of the mucous membrane of the .............. 422

W.

Watson on homicide .............. 152

West Indies, military medical report from the ...................... 65[illegible]

Western lying hospital .......... 63[illegible]

Works of John Hunter ........... 38

Wormauld's anatomical diagrams. .. 210

Wound of the ear ................ 246

Wound of the post-tibial artery...... 664

Wounded ulnar artery, case of...... 30[illegible]

Wounds, medico-legal definitions of.. 15[illegible]

Y.

Yarrell's history of British birds .... 20[illegible]

